# Paracentesis Thoracis

**Published:** 1863-02

**Authors:** 


*Paracentesis Thoracis.*—Dr. Henry I. Bowditch records the results of twelve years' experience, including 160 operations on 85 persons. He considers paracentesis as comparatively innocuous, if performed with the exploring trocar and suction pump suggested by Dr. Wyman. The difficulties occasionally ensuing are never permanent. Pain, slight dyspnœa, cough, &c., speedily passing away. He tapped one lady nine times in eight and a half months, and one elderly man, a physician, eight times in six weeks. The latter patient begged for repetition of the operation as a positive "luxury." 29 out of 75 of Dr. B.'s patients recovered apparently in consequence of the operation. The operation was performed generally whenever severe symptoms were present. In seven cases he got no fluid, but with no evil result.

He recommends to operate in every case where, whether recent or chronic, there is permanent or occasional dyspnœa of a severe character. And in *any*, even latent, cases where the pleural cavity is full of fluid, and it does not diminish after a reasonable amount of treatment.

The eligible point of operation is at the intersection of a line let fall from the lower angle of the scapula and the space between the 9th and 10th ribs. But other points may be selected. All the alleged objections to the operation, he remarks, are to him, with his experience, simply absurd. He insists that no physician has any right to allow a patient to die of the pleuritic effusion without first trying this operation. He would now as readily puncture the chest as he would draw a tooth or vaccinate a child.

The students of Rush Medical College will recollect that this practice has been strongly urged upon them (at least since the present writer became connected with the institution), and if they have had any opportunity for experience, must have come to the same conclusion with Dr. Bowditch. With a mere modicum of tact and surgical skill enough to introduce a trocar, or lance an abscess, the operation can be performed, and the patient speedily passed from a condition oftentimes of exquisite suffering to one of comparative comfort.

More than this, if performed early, the dense, false membrane is far less likely to permanently consolidate and contract, rendering the lung incapable of expansion, even after the pressure of the effused fluid is removed. In some of Dr. B.'s cases the membrane formed on the pleura costalis was so dense and firm that the trocar failed to pierce it, but pushed it inward—this would have been avoided by an earlier operation.

